# Living with advanced heart failure: A qualitative study

**DOI:** 10.1371/journal.pone.0243974

**Published:** 2020-12-14

**Authors:** Caterina Checa, Laura Medina-Perucha, Miguel-Ángel Muñoz, José María Verdú-Rotellar, Anna Berenguera

**Affiliations:** 1 Fundació Institut Universitari per a la recerca a l'Atenció Primària de Salut Jordi Gol i Gurina (IDIAPJGol), Barcelona, Spain; 2 Primary Healthcare Centre Dreta de l’Eixample, Barcelona, Spain; 3 Departament de Pediatria, Obstetricia i Ginecologia i Medicina Preventiva, Universitat Autònoma de Barcelona, Bellaterra, Spain; 4 Institut Català de la Salut, Barcelona, Spain; Maastricht University Medical Center, NETHERLANDS

## Abstract

**Introduction:**

Information about how patients with advanced heart failure (HF) live and cope with their disease remains scarce. The objective of this study was to explore, from phenomenological and holistic perspectives, the experiences of patients suffering from advanced HF, attended at home in the primary care setting in 2018.

**Materials and methods:**

Qualitative study conducted in 4 primary healthcare centers in Barcelona (Spain). Twelve in-depth interviews were conducted in advanced HF patients, aged over 65 and visited regularly at home. We developed a purposeful sampling, accounting for variability in gender, age, and socioeconomic level. Leventhal’s framework was used to analyze the interviews.

**Results:**

Participants received insufficient and contradictory information about HF. They talked about their cognitive representation and claimed a better communication with healthcare professionals. Due to their advanced age, subjects considered their daily living limitations to be normal rather than as a consequence of HF. Gender differences in emotional representation were clearly observed. Women considered themselves the keystone of correct family “functioning” and thought that they were not useful if they could not correspond to gendered societal expectations. Cognitive coping strategies included specific diets, taking medication, and registering weight and blood pressure. Nevertheless, they perceived the locus of control as external and felt unable to manage HF progression. Their emotional coping strategies included some activities at home such as watching television and reading. Social support was perceived crucial to the whole process.

**Conclusions:**

Locus of control in advanced HF was perceived as external. Healthcare professionals should adapt emotional health interventions in patients with advanced HF based on a gender perspective. Social support was found to be crucial in facing the disease. Patients reported poor communication with healthcare professionals.

## Introduction

Heart failure (HF) disease is a clinical syndrome characterized by being both progressive and physically disabling [1]. In the United States, HF is associated with a high mortality rate, representing one in eight deaths [2].

The final stage of the disease has been defined as the presence of cardiac failure symptoms at minimal effort or rest (hypoperfusion), despite the patient receiving optimal treatment [3]. According to the New York Heart Association (NYHA) scale, advanced HF is represented in stages III and IV, with an ejection fraction less than 30%, and at least one hospitalization in the previous year due to decompensation [4].

Advanced HF presents an unpredictable course that some healthcare professionals may feel insecure treating [5]. Moreover, patients may have a falsely optimistic perception about the progression of the disease and delay end- of-life discussions [6].

Patients suffering from HF have described the condition as uncertain. They find themselves in a situation that entails unexpected acute episodes which makes them feel overwhelmed [7]. This could lead to anxiety or sadness, two emotions described by subjects as having a negative influence on their quality of life [8, 9]. It is therefore relevant to explore patients’ perceptions in order to help them manage advanced HF in their day-to-day lives [10].

A previous study found that healthcare professionals focused mainly on curative treatment, patients, however, would also be interested in discussing other issues such as end-of-life topics and management of decompensations [11].

It has also been reported that during the disease’s progression, physical, psychological, and social spheres are increasingly affected whilst the spiritual domain fluctuates as patients search for the meaning of life [12].

Gender differences have been described with HF affecting men earlier than women [13]. In addition, a number of studies have highlighted the association between socioeconomic level and mortality [14]. The authors suggest that individuals’ circumstances and living conditions, as well as the social support they receive, have an impact on how HF is experienced [15, 16].

Caring for patients suffering from a chronic condition such as advanced HF, requires considering their values and concerns, since such issues could influence their behavior in relation to the disease [17]. Nevertheless, evidence regarding advanced HF patients’ daily lives is scarce [18].

There is a lack of information regarding patients’ subjective experiences of living with advanced HF which, confirms the difficulty of healthcare professionals in meeting their needs. The objective of this study was to explore, from a phenomenological perspective, experiences of advanced HF patients, attended at home in primary healthcare setting in 2018 in Barcelona (Spain), to understand the lived experience from a holistic perspective.

## Materials and methods

### Study design and population

We conducted a phenomenological qualitative study with a descriptive-interpretative approach to explore in depth the day-to-day experiences of advanced HF patients. Such an approach was focused from a non-prejudicial perspective. To guarantee confirmability, during data analysis the researchers positioned themselves from the phenomenological reduction standpoint [19].

In this study, cognitive and emotional processes have been framed within Leventhal’s common-sense of self-regulation framework [20] and the research team positioned itself from a situative approach.

Patients’ eligibility criteria are summarized in Table 1.

**Table 1:** Summary of inclusion and exclusion criteria.

Inclusion criteria	Exclusion criteria
**Patients diagnosed with HF NYHA III and at least one hospital admission in the previous six months**^**§**^**, or grade IV**	Dementia
**Attended at home by primary care professionals**	Intellectual disability
**Aged > 65 years**	Severe psychiatric disorder
	Presence of symptoms that could impede describing the experience

^§^ Bjork JB, Alton KK, Georgiopoulou VV, Butler J, Kalogeropoulos AP. Defining Advanced Heart Failure: A Systematic Review of Criteria Used in Clinical Trials. Journal of Cardiac Failure. 2016; 22(7):569–77.

HF: heart failure. NYHA: New York Heart Association.

### Sampling and participant selection strategy

The study was conducted in Barcelona (Spain) in four primary healthcare centers (PHCC) located in four different neighborhoods which reflect the city’s variability in socioeconomic status [21]. The characteristics of each PHCC are depicted in Table 2.

**Table 2:** Socioeconomic characteristics of primary health care centers.

Primary health care center	Population	Deprivation index^§^
**Dreta Eixample**	47594	14.91
**Sant Martí**	41158	44.52
**Clot**	25866	35.13
**Sant Elies**	33727	3.33

^§^Department of Health of Catalonia, Spain. Webpage: http://observatorisalut.gencat.cat/ca/observatori-sobre-els-efectes-de-crisi-en-salut/indicador_socioeconomic_2015/.

We developed a purposeful sampling based on a description of potential participant characteristics extracted from medical records to obtain optimal variety and richness in their discourses. The following variables were taken into account: gender, age, and socioeconomic status. In addition, variables related to the stage of the disease (NYHA III / IV), next of kin, number of household cohabitants, and degree of family relationship was collected. Informative richness and data saturation was achieved.

In each PHCC, a nurse coordinator was assigned to enhance communication between the center and the research team. From February to April in 2018, the coordinator identified patients who met the inclusion criteria and informed the principal investigator. The latter confirmed their suitability for the study and contacted the potential participants by telephone.

In the first contact, the principal investigator explained the study to the patients and emphasized its voluntary and anonymous character. Time was allowed for them to decide whether or not they wished to take part. Once the patients had agreed, appointments were arranged at their homes and informed consents signed before starting the interviews. All the contacted participants agreed to participate in the study.

### Data collection

All interviews were moderated by the same investigator from the research team, guaranteeing both credibility and homogeneity. The interviewer used open, non-directive questions that were adapted to the participants‘ command of language. The topic guide included six main topics (Table 3): information about the disease, relationship with healthcare professionals, daily life, social support, mood, and the future.

**Table 3:** Interview guide topics.

*Please*, *we would like to listen to your opinions and experiences on*:	
Information about the disease	*When was the first time you heard about heart failure*?
	*Who told you*?
	*What information did they give you regarding the illness and how did you feel*?
	*What did you think at that time*?
Relationship with healthcare professionals	*How is your relationship with healthcare professionals*?
	*Do you feel well informed about heart failure*? *Which professional do you think helps you the most in this*?
	*How often do professionals come to see you at home because of your health problem*?
	*What recommendations do professionals give for the treatment of heart failure*?
	*What are the main difficulties in following the therapeutic recommendations prescribed by primary care professionals*?
	*What do you think the healthcare professional can do more in relation to heart failure*?
	*Do you feel that health professionals care about your heart failure*?
	*How would you like your primary care professionals to help you*?
	*Do you find something missing in the healthcare attention*?
Daily life	*Does your heart disease affect your daily life*? *In which sense*?
	*What activities or strategies help you to live with heart failure in your daily life*?
	*What activities recommended by healthcare professionals are easiest for you to follow*?
	*Do you think you can do something to improve your illness*?
Social support	*Do you think heart failure affects you in your family and social relationships*?
	*In what sense has it affected you*?
	*How important is social support for you*?
	*What strategies do you use to try not to let this not affect you*?
	*How do you feel about that*?
Mood	*How do you feel about the disease*? *Can you describe it to me with a feeling (adjective)*?
	*How do you go about trying to feel better*? *Do you find space to relax*?
	*Do you think you have learned to take care of your illness*?
Future	*How do you imagine the future with the disease*?
	*What do you think can happen*?
	*Has anyone talked to you about this*?

During the interviews, the participants had the opportunity to discuss new topics that they considered relevant to their daily experiences.

The research team started perceiving data saturation after 9 interviews. Finally, twelve interviews were conducted achieving data saturation. The interviews lasted between 20 and 60 minutes and took place at the patients’ homes. Interviews were audio-recorded, transcribed verbatim, and contextualized using field notes.

### Analysis

**Fig 1:**
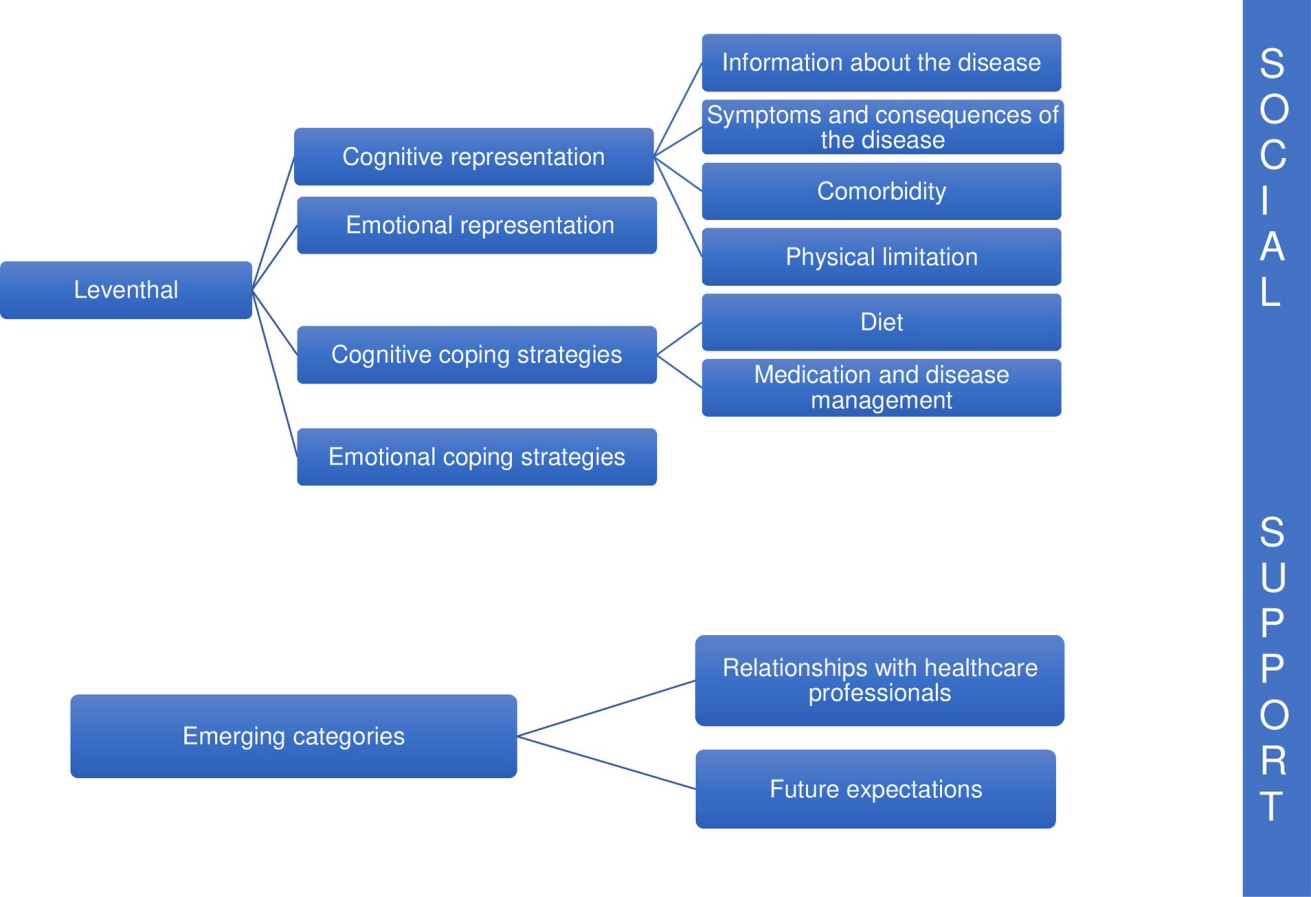
Category tree figure.

Thematic content analysis was used to analyze the interviews, employing steps developed by Berenguera et al. [22]. First, the interviews were read and re-read to attain a pre-analytical insight. Data were then analyzed based on the following steps: a) identification of the main topics, b) fragmentation into units of meaning, c) text codification with mixed strategy: using Leventhal‘s framework [20] and emerging codes, d) creation of the categories, e) analysis of each category, and f) results elaboration. All members of the research team were involved in the data analyses and consensus was sought for each step. The new framework was discussed with the research team members until a consensus was achieved (triangulation of data). This framework is provided in Fig 1.

### Ethical considerations

The study followed the tenets of the Helsinki Declaration and Good Clinical Research Practice [23]. The nurse provided information about the aim and procedures of the study. All participants signed a written informed consent form before starting the interviews. Confidentiality and anonymity were ensured by assigning to each participant a code which was used to identify the transcripts. The project was approved by the Ethics and Clinical Research Committee of the Primary Care Research Institute Jordi Gol (4R17/010). The audio recordings will be securely deleted 5 years after study completion.

### Framework

The Leventhal framework conceptualizes individuals as active “solvers” of their health problems. Initially, individuals tend to receive diagnosis as a threat to their health, and as a result, develop cognitive and emotional representations. Such representations lead to attempts at self-regulation that may involve different coping strategies that interact with each other. Individuals then assess the consequences of their actions and self-regulate their response in a system of continuous feedback [20].

Additionally, the research team was positioned from the situative approach. This is characterized by their were being involved in the phenomenon, learning from it progressively. The learning process is gradually improved due to the researchers’ desire to participate in the investigation and increased appearance of feelings of belonging to the study phenomenon [24].

## Results

Participant characteristics and codification are available in Table 4. Results are presented following Leventhal’s framework (cognitive and emotional representations, cognitive coping and emotional strategies, and social support) and emerging codes (healthcare professional relationship and expected future).

**Table 4:** Clinical and social characteristics of participants.

Area	Deprivation index	Gender	Age	Stage of the NYHA classification	Academic level		Next of kin	Number of cohabitants^§^	Verbatim code
**Dreta Eixample**	14.91	Women	81	III		University	Husband	2	MM81
			88	III		Secondary	Son	3	LT88
		Men	86	IV		University	Wife	2	JB86
			89	III		Elementary	Wife	2	ES89
**Sant Martí**	44.52	Women	77	III		Secondary	Daughter	1	AR77
			79	IV		Elementary	Husband	2	JM79
		Men	70	IV		Elementary	Wife	4	AM70
			82	IV		No studies	Wife	2	JM82
			92	IV		No studies	Daughter	3	VS92
**Sant Elies**	3.33	Women	74	III		Elementary	Daughter	6	CG74
		Men	76	III		Secondary	Son	2	RP76
**Clot**	35.13	Women	92	IV		No studies	Daughter	2	DA92

^§^Total number of cohabitants including the participant. NYHA: New York Heart Association.

### Leventhal framework

#### 1. Cognitive representation

Cognitive representations refer to how patients first experienced HF, and its consequences, with respect to their daily life (symptoms, comorbidity, and physical limitation).

*1*.*1 Information about the disease*. The participants explained that they were diagnosed with HF after a long period of decompensations. During the interviews, it was observed that they tended to normalize HF symptoms, and even referred at them as a consequence of aging. The participants first received information about HF from healthcare specialists while in hospital for an exacerbation. They claimed that both information and guidance on how to manage the disease in daily life were scarce. Details provided included the possibility of an intervention which was understood as a good sign and that the heart could be “repaired”.

The patients generally felt that healthcare professionals employed a wide range of technical language that made it difficult for them to understand full explanations. As a result, they tended to adopt a passive attitude towards the information they were receiving and were often excluded from any dialogue with healthcare professionals, so there was no option for a shared decision-making process.

*JM82 “They say I have heart failure… but they talk to each other [between healthcare professionals] … they said nothing to me… they auscultate my back and made their conclusions*, *I don’t know…*”

The locus of control in advanced HF was perceived as external. Patients sought an explanation for their illness, and the majority considered the contributing factors to be beyond their control. Such a situation led to feelings of desperation and impotence. Only two participants referred to the fact that external causes could lead to HF: hereditary issues and a poor lifestyle.

*CG74 “What I have is a hereditary disease… we all suffer from the heart in my family*, *we get the tests and half of my family is ill… our ancestors*? *Could have left us money but no*! *They left us diseases*!”

*DA92 “I don’t know how I can be ill… I always eat well*, *because I try to take care of myself … I had sugar on my blood and now I don’t… I used to have cholesterol and now I don’t*… *I was on a diet… I don’t know why I have this*”

*1*.*2 Symptoms and consequences of the disease*. Patients were provided with descriptions of various techniques to treat HF such as catheterization, pacemaker implants and a cardioverter-defibrillator. They did not, however, consider such interventions as forming part of their chronic treatment but merely occasional measures to improve their condition. In fact, they did not receive any explanations about which intervention was the most appropriate and its relevance.

Furthermore, the participants felt unable to manage decompensations. Advanced HF involves many hospitalizations between stable periods, and patients admitted to sometimes delaying admission, or even refusing to attend, as way of rebelling against the disease and temporarily breaking treatment adherence.

*JM82 “I have to go a lot to the hospital and stay there for days*. *Every two or three months I go… Now I am… I don’t go now because I am a stubborn person because if not*, *I would be in the hospital again*. *Now I feel like I should be in the hospital*”

Suffering from HF involves everyday symptoms such as chest pain, breathlessness, edema, and marked tiredness that participants sometimes referred to as part of aging. All of them, however, mentioned pulmonary congestion as aggravating baseline symptoms.

During the interviews, symptoms were highlighted as potentially representing a considerable emotional burden.

*JB86 “When I walk I get tired… and I thought at the beginning that I couldn’t live like this… and of course*, *it was the heart what was damaged… the pacemaker helps a lot*, *I have had it from twelve years or so*, *but I still feel tired*”.

*1*.*3 Comorbidity*. Besides advanced HF, participants talked about other conditions that worried them and had a negative impact on their daily lives. Almost all the patients mentioned diabetes and most of them renal failure. All the comorbidities referred to were considered as part of the aging process and not lifestyle factors. Participants explained interactions between diseases and treatments that made it even more difficult to cope with the whole experience.

*ES89 “As you get older*, *the diseases appear… because on top of that I have diabetes but type II… then they (healthcare professionals) saw that my kidney is not working normally … and… if I take diuretics and… this is constantly happening to me… then I found that it kills my kidney… and if someday I need cortisone this unevens sugar*! *So*, *you have to calculate the insulin… I always check my sugar level… one day my wife found me unconscious and my sugar level was 26*!”

*1*.*4 Physical limitation*. During the interviews, all participants described difficulties in carrying out their daily activities and remarked this was their principal day-to-day limitation. They commented that they depended on their family or caregivers to help them with basic tasks such as bathing and dressing.

Furthermore, there was a marked concern among women about limitations in carrying out housework, and they emphasized their wish to gain physical independence in order to manage it better. The burden of physical limitations was different for men since they referred to being adapted to the disease and, moreover, being used to be cared by others.

Physical limitations in both genders were generally attributed to aging and not necessarily to the disease itself. However, patients sometimes felt an increased baseline tiredness which they ascribed to HF exacerbation and were able to distinguish the periods they occurred.

The discrepancy between health recommendations and the participants’ own physical capacity was highlighted when they commented that some of the non-pharmacological recommendations (maintaining physical activity and weight themselves) were impracticable due to baseline symptoms and physical limitations.

*AR77 “… I am also older*, *so I am more tired… not for just a walk…no*, *but at home*, *there are a lot of things that I would like to do and I can’t*, *for example cleaning mirrors*, *the kitchen*, *the bath… professionals told me to walk*!!! *hahaha I can’t*!!!” *(Woman)*

*VS92 “Because don’t move too much*… *I do*… *I wash*… *I shower*… *(coughs)*… *Slowly I shower*, *I don't move from here*… *There are people who cannot have a relaxed life*, *of course*… *A person who cannot stop from moving*… *But if I have no need*! *why should I go for a walk*? *Or do things*? *I have no need of this*” *(Man)*

#### 2. Emotional representation

Emotional representations refer to patients’ emotional experiences related to living with advanced HF.

For the participants, advanced HF had repercussions on their mental health. All the women experienced sadness and resignation when diagnosed. They described how they felt unfortunate, and were concerned about the future and how their disease could affect their roles within the family. The female participants also admitted to feeling depressed and lonely, and sometimes suffering from anxiety. On the other hand, men appeared to be more relaxed and even optimistic about being cured.

*MM81* “*so at first I felt bad*, *well very bad… very sad… I thought a lot about this… why it happened to me*?*” (Woman)*.

*AM70* “*…well*, *it happens to me (think a lot at night) … because all my family goes to bed and in that moment I feel alone I think… it is not depression*, *I think it is not… but it is just think and think… but then I feel I cannot live like this*! *I cannot live with fear because in this case it will be better to die*! *so generally I am calmed…” (Man)*.

*DA92* “*I'm getting worse and worse… I have depression*! *I am exhausted with everything*. *I used to do many things at home*, *dresses*, *quilts*… *now I do not even want to do anything anymore*, *how do I feel*? *Bad*… *I'm not good doing anything*, *I used to do everything*! *Now I have nothing left” (woman)*.

Both genders referred to frustration, feeling they were a burden for others and dependent on them for basic daily activities.

Emotional and cognitive representations are not necessarily independent from each other, and could be in constant interaction to create the representation of the disease.

#### 3. Cognitive coping strategies

This section describes cognitive coping strategies to improve physical well-being through diet, medication, and disease management.

*3*.*1 Diet*. In order to cope with the disease and prevent decompensations, some participants mentioned they could partially avert congestive symptoms with diet. This coping strategy was perceived as simple adherence to healthy food guidelines. The participants followed general recommendations, a hypo-sodic diet, and water restriction. One participant felt these were too strict since, in addition to HF guidelines, he followed others for his comorbidities.

*AM70* “*Well*, *especially the diet*… *because in the last analysis*, *besides the sugar*, *there was also the salt*… *they are suppressing everything*! *This is too much*. *Sometimes they even ban beer without alcohol…”*

*CG74* “*I have to take care of food*, *that’s normal*… *I cannot say that they do not tell me*… *because they have told me 200 times*… *that I should not eat salt*, *that I should eat vegetables*, *fruit*… *I should not eat sausages*… *it is easy for me*, *I eat everything*… *for example*, *I made a stew*, *so I cook it light with only vegetables*.*”*

*3*.*2 Medication and disease management*. Another way to prevent congestive symptoms was through medication. All participants said that they had to cope with this issue, and highlighted the fact that they had to deal with constant changes in medications and doses. They referred to diuretics as a common drug for HF, and even though intake was generally easy, considerable discomfort could arise due to the need to frequently urinate.

*VS92* “*You have to endure*… *you cannot make any effort*, *having a peaceful life… you go day by day because if you make some kind of effort you will feel worse*! *And now the pills… they (professionals) change a lot my medication… I have had a really bad year… They say one at first*, *then other… they drive me crazy*! *For the moment*, *I take a lot of pills and that’s all”*

For some participants, having their blood pressure taken was a crucially objective sign of good disease management, as was weight measurement when possible. Only one participant talked about quitting unhealthy habits such as smoking as a first step for good HF management.

All of them sought a quiet life with no physical effort due to baseline symptoms.

*ES89* “*Well I take this as a routine… I am a very pragmatic person… I wake up*, *I weigh myself*, *I take the pills… the diuretic one first…that’s a recommendation*, *I measure my blood pressure*, *I measure my sugar levels… I have to have everything under control*, *sometimes it gets wrong in one way*, *sometimes to another…”*

#### 4. Emotional coping strategies

Emotional coping strategies refer to how advanced HF patients cope with emotions and improve their daily lives.

Advanced HF affects had repercussions in mental health, and all the participants emphasized some strategies to enhance it. Such strategies were based on the completion of domestic activities (watching television, reading…) positive thinking and social relationships. They said that these activities allowed them to keep their minds concentrated and focus attention away from the disease.

*AR77* “*… I try to speak with my friends*, *I have friends I speak with*, *they come here*, *well*, *she is widow now too… before me… and we talk a lot… I get distracted… when I think too much I switch on the TV and I watch it or I do an alphabet soup… I try not to think too much… I take a book and read… I try also not to worry my family…they call me “mama*, *how are you*?*” “Fine*, *dear*…*”*

The participants also reported that being grateful, for example, for living in their own homes and maintaining social relationships, provided benefits with respect to coping with emotions.

*VS92* “*What can you do*? *Be angry*? *Yes… hahaha… What I have to do is be grateful for being here*! *There are many people that cannot explain this and I am here now*. *You have to be always grateful*. *It is not worth to think too much*… *you have to be adaptable”*

Some patients added that minimizing HF symptoms and consequences in family relationships helped divert attention from their health and maintain better communication with their relatives. For all of them, social support was crucial in coping with emotions.

A few explained that religion alleviated their suffering and provided acceptance. They explained that God helped them to overcome their daily lives by bringing peace of mind. For some participants religion was very important, and they gained comfort practicing its rituals.

*MM81* “*this suffering*… *what I am suffering now I do not know if many people would endure it*… *and I have faith*… *because if not*… *you will be buried*… *God helped me because I was not well*, *I did not do anything wrong in life*…*”*

#### 5. Social support

Living with advanced HF is a strenuous process that has both physical and psychological consequences. From the interviews, social support was found to be a transversal category that played a key role in the patients’ experiences of HF.

All the participants reported that good social support brought them relief and strength. In fact, those who felt they did not have enough expressed stronger feelings of depression and poorer quality of life.

JM79 “*Well*, *my sister helped me a lot*. *You have to think that she came here every day*, *and she had her job*… *And I came every day to wash myself*, *to dress me*… *Well*, *to dress me*… *To change me this*, *to change that*… *Yes*, *she*… *We must be very grateful to her*. *Because*, *who did it to me*?*”*

Social support came mainly from the participants’ families although some subjects said that friends were also important. Despite having more friends prior to diagnosis, the quality of the relationships was greater at the moment of the interviews. For most participants, being ill had led to generating stronger links with some people and the loss of other friendships. They valued meaningful relationships and found them helpful in coping with all aspects of the disease (both cognitive and emotional).

The patients also considered formal caregiver support as essential in order to perform basic daily living.

*LT88* “*I will need 2 or 3 hours more per week of help (carers)*, *more than anything for the company*! *Because sometimes these girls that come*, *when we have finished showering or fixing my feet… I tell them*, *look we will talk for a while*! *And sometimes when my grandchild comes*, *I tell him*, *hang me that*, *do me that*… *and that also affects me because*, *well*, *if you want to do something*, *you have to wait for someone*, *you should always have someone*.… *that helps you*, *that's what has depressed me quite a lot*, *you know*?*”*

#### 6. Emerging categories

Categories were identified that did not fit within the Leventhal Model. These were related to relationships with healthcare professionals and participants’ expectations for the future.

*6*.*1 Relationships with healthcare professionals*. During the interviews, the participants emphasized the importance of a trusting relationship with the healthcare professionals. They valued such qualities as kindness, affection, honesty, and compassion. In general, the participants felt that primary healthcare professionals were concerned about their health status. In periods of stability, however, the patients perceived that they were less involved with the healthcare system, which led to a feeling of loss of control with respect to the disease.

*AR77* “*Time ago professionals took a lot of care*, *but now they see that I am very stable*… *And then I do not*… *I do not think they are very worried*! *“*

Only one participant reported having a negative relationship with primary healthcare professionals and little contact. This participant highlighted the fact that a trusting relationship with primary healthcare professionals was more valuable than medication. The same individual remarked on the importance of the longitudinal characteristics of the primary care services provided by scheduled home visits.

*JM82* “*Because they have told me 20 times that the doctors would come and nobody comes here*. *No one comes*! *Lie*! *They tell you there in the hospital*, *or in the outpatient clinic*, *whatever*… *But here nobody comes*! *I believe*, *I have always believed*, *I have always thought that good personal treatment would cure more than pills*. *They would have to treat you with more humanely*. *Sure*, *I don’t have studies to explain this*…*I think it but I cannot explain*… *I am very basic*, *illiterate*… *I can not*… *Well*, *that would have to be more human*, *and give a more human treatment to the patient*, *the patient*… *Sometimes we are looking forward to a humane visit”*

In relation to specialist healthcare professionals in hospital, the participants had a trusting relationship with their cardiologist. They added that having a contact phone number to reach a healthcare professional provided comfort. Advanced HF patients found the emergency departments to be chaotic, noisy, uncomfortable, and unsuitable for their needs. They preferred scheduled options such as planned hospital admissions or home treatment.

All participants reported receiving home visits from primary healthcare professionals who helped them manage the disease. Nevertheless, they perceived that the most specialized HF care was provided by a cardiologist during scheduled appointments, and when admitted to hospital wards during a decompensation.

*6*.*2 Future expectations*. The participants also wondered about their future and found it predictable. Although they claimed that no healthcare professional had explained to them how they would feel, and the care they would need, they said they did not expect to be cured and were aware they would be more physically dependent. They also understood physical dependency as a normal state of aging.

*MM81* “*a future*… *very long future I do not have it*, *dear*. *A very long future I do not expect*… *I have to ensure that in the future I will not lose joy*… *do not miss*… *the future I cannot say*…*I have to think about living the day to day*…*Every day I will be building a bit of that future*… *Professionals don’t talk to me about it because*… *what can they tell me*?… *What can you tell me*?

In addition, all the emotional coping strategies such as positive thinking, fostering meaningful relationships, and the completion of household activities played a key role in facing the future.

Only one participant avoided receiving information about the future, as a way of coping with her fear of an unfavorable progression of the disease.

*JM79* “*I can’t imagine my future and I don’t ask information about it… you know what*? *I don’t want to know what is going to happen”*

## Discussion

### Summary of main results

Participants described receiving insufficient and contradictory information about HF and referred to the fact that diagnoses was usually made during exacerbation in emergency rooms. For most of them, limitations in daily living were perceived as normal given their advanced age rather than being attributed to their condition. Since being older is usually associated with suffering and physical deterioration, the participants felt resigned to their situation, and normalized their deteriorated health status.

Regarding emotional representation, gender differences were clearly observed. Women considered themselves as playing a crucial role in the "proper functioning" of the family.

The participants in our study talked also about cognitive representation after diagnosis, and claimed they needed better communication with healthcare professionals. Cognitive coping strategies included following a diet, taking medication, and registering some objective measurements such as weight and blood pressure. Despite such strategies, the patients felt that the locus of control was external and considered themselves unable to manage HF progression.

Emotional coping strategies to manage HF included some activities at home such as watching television and reading.

Social support was perceived as crucial to the whole process in coping with the disease.

### Discussion of the main results

Early diagnosis is known to be crucial in order to start proper treatment in HF patients [25]. All our participants had been diagnosed after a considerable period of time during which they had normalized HF symptoms and attributed them to aging. Diagnosis normally takes place in hospital emergency departments when patients need to be treated as a consequence of an exacerbation of the disease [26] as was the case of our sample.

Such a context does not always guarantee the necessary conditions for fluid communication, patients can feel overwhelmed since they do not fully understand the extent of their condition [27]. Moreover, it has been demonstrated that the way in which physicians provide information is crucial since this has an impact on understanding and treatment adherence [28]. We therefore consider it important to create specific protocols to communicate diagnoses in emergency department settings, and specifically for HF in acute decompensation situations, since patients often resort to the emergency rooms without a previous diagnosis.

With respect to developing such protocols, it should be noted that patients have reported preferring to receive a diagnosis in quieter and more comfortable venues than emergency rooms. A specific location should therefore be allocated to deliver this type of news so that patients are in a calmer environment and thus feel freer to ask questions and become more involved in the health process [29].

Regarding our participants, following diagnosis they did not usually have well established follow-up appointments with primary healthcare services. Consequently, they resorted to emergency services when they felt unwell. It has been observed that patients lacking primary healthcare follow-up have a higher probability of being re-admitted to hospital [30]. Such a situation could be aggravated by poor coordination between hospitals and primary healthcare professionals leading to misinformation in primary healthcare regarding the patient’s current health status after hospital admission [31]. Primary care home-schedule appointments for patients in stable periods need to be set up in order to improve communication and promote empowerment of advanced HF patients.

Although primary healthcare is more accessible than other healthcare settings, and takes into account how patients feel [32], subjects feel safer in hospitals [33]. This could be due to the fact that they tend to prefer specialized care to manage their decompensations. Even though primary care offers a better holistic approach when evaluating overall health, hospitals provide a wide range of complementary measures and techniques. Nevertheless, we found that patients tried to be admitted as little as possible because of the disruption to their lives [34].

Locus of control is a concept used to represent the degree of influence that individuals perceive they have over their health. The internal locus of control refers to subjects thinking that their personal decisions, for instance, diet, could influence their health. The external locus is the belief that one’s health is out of one’s control [35] which can lead to a wide range of negative emotions.

The locus of control in our participants was mainly perceived as external since they felt they had no control over the progression of the disease. They considered the course of the disease and its decompensations would not be modified by lifestyle changes, for instance, a hyposodic diet. Apart from a study in HF patients which reported that patients felt able to influence the course of the disease [36], there is no further information about the locus of control in advanced HF patients.

An external locus of control could also be related to a lack of empowerment which is not always facilitated by healthcare professionals. Our participants explained that some recommendations given by health professionals (e.g. physical exercise) did not correspond to their current status and could not be performed. This could diminish the relationship of trust with the professionals and lead to a decrease in treatment adherence [37]. We consider it crucial that recommendations be adapted to the patients’ functional status.

In this context, “virtual communities of practice" have been used among health professionals to share experiences about patients and better understand how to empower them. Such communities have been well accepted and are a way of facilitating peer learning [38]. It could be useful to implement this tool in daily practice.

Social support plays a key role and contributes to disease management [39]. Caregivers are considered to be crucial following hospital discharge, they are a source of comfort, advocating, and even acting as a mediator between patient and healthcare provider [40]. We found social support to be critical in motivating patients to cope with the disease and observed a strong link between lack of social support, sadness, and a worse perceived health status [41]. It is plausible to think that, in addition to the operational help that social support can provide, patients could receive informal emotional support from caregivers, family and friends. In this regard, it was found that patients with good social support had better levels of mental health and self-care [42].

It is necessary, therefore, to create programs to help individuals with limited social support improve their levels of adherence, mental health, and self-care. Indeed, a specific intervention based on coping strategies such as relaxation training, cognitive restructuring, and problem solving in patients with lack of social support has shown to be not only cost-effective, but also instrumental in improving quality of life and reducing depression [43].

In terms of emotions, we found that women expressed feelings of sadness, loneliness, and even desperation caused by their marked physical limitations. Such situation is probably due to their socially assigned caregiving/reproductive roles which cannot be fulfilled due to the basal symptoms and physical decline of advanced HF [44]. Whilst it is accepted by both genders that men are weaker when they get sick and become more dependent [45], our male participants claimed to be relaxed and well-adapted to the disease. Such a difference could be partially explained by the tendency of men in patriarchal societies to want to appear stronger and not express emotions [46]. All of which could result in depressive feelings not being expressed thus leading to a perceived “good” adjustment to illness. Nevertheless, we are not aware of any previous authors examining in depth emotional gender differences and masculinity in patients with advanced HF and its impact in healthcare.

These gender-emotional differences have, however, been studied in other conditions such as chronic pain. The authors concluded that there is a gender bias in treatment management with women being generally undertreated [47]. Specific training regarding gender perspectives for health professionals could provide a better healthcare approach to emotions.

This study on advanced heart failure patients provides new information concerning the experience of living with the disease, and could help healthcare professionals to understand needs, preferences, and expectations in order to improve the care provided.

## Strengths and limitations

The main strength of this research is that we could explore in depth the experience of patients living with advanced HF. Furthermore, the interviews held at their homes provided the opportunity to collect data in their context. We believe this to be of value as narratives can be richer and more truthful regarding the patients’ experiences. In addition, the analysis was performed by a multidisciplinary research team and data were mapped based on a theoretical model.

Our study was based on theoretical-methodological and technical concordance in order to properly achieve the main objective. The rigorous procedures employed (triangulation, data saturation, and the characteristic flexibility of qualitative methodology) ensured the validity of the results. Caution is needed when extrapolating these results to other populations. Nevertheless, we consider that the experience of advanced HF in our participants may be transferable to those in most developed countries. Although the sample was only 12 participants it was sufficient to obtain results about their experience. In phenomenology, small samples can give considerable information regarding individuals’ perceptions. Ours sample was taken from an elderly population so it was common for them to present comorbidities. We believe that this should be taken into account when understanding their experiences of health and illness as a whole.

## Conclusions

Patients with advanced HF consider the symptoms and consequences of the disease as part of the process of aging more than the disease itself. The locus of control of the disease was perceived as external because participants felt that they could not influence the course of HF.

We found differences in emotional representation between genders. Women referred more to depressive symptoms while men claimed to be calmer and even hoped to be cured. Healthcare professionals should adapt emotional health interventions in patients with advanced HF based on a gender perspective.

Regarding cognitive coping strategies, patients found treatment generally easy to follow, but some lifestyle recommendations could not be performed due to the patients’ physical limitations. In all the process, social support was considered crucial when confronting advanced HF.

Participants reported poor communication with healthcare professionals.

## Clinical implications

- Patients believe that the information provided by professionals is scarce. Therefore, it is necessary to involve patients in care and dialogue to provide all the necessary guidance. Furthermore, patients suffering from advanced heart failure value a trusting communication and optimum relationships with healthcare professionals.
- Since social support is crucial for patients with this disease, it should always be taken assessed in clinical evaluations.
- Professionals should take into account the emotional differences between genders and incorporate mental health evaluations.
- Since the locus of control is perceived as external, professionals should encourage patients to take control of the disease and give them recommendations they feel they can perform.

## Further research

- In view of the fact that patients may feel excluded from the care process, we consider it crucial to develop patient and public involvement in research (PPI) strategies.
- More research should be done regarding the locus of control and its repercussions in patients with advanced HF.
- Emotional differences between genders should be investigated in depth.
- Given that HF suffering and symptoms are considered normal in the elderly, it would be interesting to investigate the impact of this perception on the disease.
